# Are the Results of Late Heart Re-Transplantation Influenced by the Time Interval from Primary Transplantation?

**DOI:** 10.3390/jcm15020564

**Published:** 2026-01-10

**Authors:** Andrea Lechiancole, Gregorio Gliozzi, Stefano Copetti, Chiara Nalli, Concetta Di Nora, Giovanni Benedetti, Sandro Sponga, Uberto Bortolotti, Ugolino Livi, Igor Vendramin

**Affiliations:** 1 Cardiothoracic Department, Azienda Sanitaria Universitaria Friuli Centrale, University Hospital of Udine, 33100 Udine, Italy; gregorio.gliozzi@asufc.sanita.fvg.it (G.G.); copetti.stefano@spes.uniud.it (S.C.); chiara.nalli@asufc.sanita.fvg.it (C.N.); concetta.dinora@asufc.sanita.fvg.it (C.D.N.); giovanni.benedetti@asufc.sanita.fvg.it (G.B.); sandro.sponga@asufc.sanita.fvg.it (S.S.); cardiochirurgia@asufc.sanita.fvg.it (U.B.); ugolino.livi@uniud.it (U.L.); igor.vendramin@asufc.sanita.fvg.it (I.V.); 2 Department of Medicine, University Hospital of Udine, 33100 Udine, Italy

**Keywords:** heart transplantation, coronary allograft vasculopathy, heart re-transplantation

## Abstract

**Background**: Heart re-transplantation represents an effective option in selected patients with graft failure. Although the results of heart re-transplantation have improved in recent years, little is known about the outcomes in patients requiring late (>1 year) re-transplantation or about the effects of prolonged exposure to immunosuppression on multiorgan function. **Methods**: Among all the heart re-transplantations performed, from November 1988 to April 2025, a total of 30 patients underwent late re-transplantation. Since literature data on late re-HTx are generally limited to those performed within 20 years, patients were divided according to the interval from primary to re-transplantation: <20 (Group A) or ≥20 years (Group B). **Results**: Group A included 17 patients re-transplanted after a median time of 15 years, and Group B 13 patients after a median time of 23 years. Group B patients were more commonly affected by severe chronic kidney disease and received more combined heart and kidney transplantation. Overall survival at 1, 5 and 10 years was 80%, 68% and 60%, and did not differ between Groups A and B (*p* = 0.5). However, Group B recipients were more commonly affected by malignancies (*p* < 0.01). Compared to primary heart transplantation in the same population, re-transplantation was associated with higher, albeit not statistically significantly, rates of infections, grade ≥ 2 rejections and malignancies. **Conclusions**: Late heart re-transplantation is associated with satisfactory outcomes and could be effectively considered in patients with late graft dysfunction. However, the prolonged exposure to immunosuppression requires particular attention in early pharmacological management as well as a close follow-up, especially in patients requiring heart re-transplantation after >20 years.

## 1. Introduction

Heart re-transplantation (re-HTx) represents a feasible therapeutic option, indicated for patients with severe dysfunction of the transplanted graft. Reported for the first time in 1977 [1], re-HTx accounts for only 2–4% of all HTx procedures performed annually [2]. In fact, there is widespread concern regarding re-HTx because of the limited available donor pool and the higher mortality and morbidity compared to primary HTx reported in the early experiences [3,4,5].

Despite attempts to define the criteria for an ideal re-HTx, many centers are still reluctant to adopt this practice. Indeed, according to the Italian National Center for Transplantation (CNT), between 2002 and 2021, only 74 patients (<2%) underwent re-HTx in Italy [6]. Most currently available data on re-HTx are derived from large international registries reporting dismal results if re-HTx is required within 1 year from primary HTx [7,8,9,10]. However, outcomes could be severely compromised by the prolonged immunosuppressive therapy and its long-term effects on multiorgan function, but data on late re-HTx (>1 year) are generally limited to those performed within the first two postoperative decades [2,3,4,5,10,11] after the primary HTx.

To provide further data on this important issue, we report here our single-center experience with the aim of assessing the risk of mortality and morbidity in patients requiring late re-HTx, with particular attention to those who received a second graft beyond the second decade from primary HTx.

## 2. Patients and Methods

### 2.1. Patient Data

From November 1988 to April 2025, a total of 729 HTx and 35 re-HTx (4.8%) were performed; 30 patients undergoing late re-HTx were included in this analysis. The study was approved by the Institutional Review Board (Clinical Registration Number: 149/2025 of 12 May 2025), and informed consent was waived due to its retrospective nature.

Data were prospectively collected in our institutional dataset and retrospectively analyzed. Follow-up was 100% complete and obtained through periodic ambulatory visits. In order to assess the impact of the time interval on the results of late re-HTx, patients were divided into two groups, Group A (17 patients, <20 years from initial HTx) and Group B (13 patients, ≥20 years from initial HTx), analyzing and comparing baseline data and outcomes.

As a secondary endpoint, complication rates after first transplant and re-transplant were evaluated in the overall population.

### 2.2. Definition of Terms

Extended-criteria donor characteristics were considered those derived from the OCS-Heart Expand Trial [12]. Primary graft dysfunction (PGD) [13], chronic kidney dysfunction (CKD) stages [14], rejection episodes [15] and coronary allograft vasculopathy (CAV) [16] definitions were those currently accepted and available from the most recent guidelines. Chronic graft dysfunction was defined when occurring in the absence of CAV or acute rejection.

### 2.3. Surgical Technique

The St. Thomas II solution was utilized for inducing cardioplegic arrest in all donor hearts retrieved. Graft preservation techniques included traditional ice-cold storage, ex situ normothermic perfusion with the Organs Care System (OCS; TransMedics Inc., Andover, MA, USA) and controlled hypothermic storage with SherpaPak (PSP; Paragonix Technologies, Inc., Cambridge, MA, USA) devices [17]. Donor grafts were implanted using the bicaval technique, irrespective of the technique adopted during the primary HTx.

Associated kidney transplantation (KTx) was considered in all patients requiring chronic hemodialysis, and in those with stage ≥4 CKD in whom renal impairment was deemed not reversible after re-HTx [18]. The choice between a simultaneous or staged KTx was based on the hemodynamic stability and clinical status of the patient.

### 2.4. Statistical Analysis

Continuous variables are expressed as mean ± standard deviation (SD) or median and interquartile range (IQR), according to the data distribution, and were analyzed using the Shapiro–Wilk test to verify a normal distribution. Categorical variables are presented as absolute numbers and percentages. Student’s t test or Mann–Whitney U-test was used to compare continuous variables between groups. Comparison of categorical variables was performed by chi-squared analysis or Fisher’s exact test, as appropriate. Kaplan–Meyer survival curves were created and compared with the log-rank test.

Univariable and multivariable Cox regression models were used to estimate factors associated with overall survival after verifying the proportional hazards assumption. Multivariable analysis included all variables statistically relevant in univariable analysis (*p* < 0.05), considering the number of events and potential collinearities.

All statistical analyses were performed using the Statistical Package for Social Sciences (SPSS, version 24) program (Chicago, IL, USA).

## 3. Results

### 3.1. Baseline Patient Characteristics

Thirty patients required late re-HTx within a median time interval of 17 years (range 4–37 years, IQR 12–20 years). Among these, late re-HTx was performed after a median interval of 15 years in Group A (range 4–19 years, IQR 7–17 years) and 23 years in Group B (range 20–37 years, IQR 21–25 years). Other baseline clinical characteristics are detailed in Table 1.

Primary HTx was performed according to the bicaval or biatrial technique in 15 and 2 (88% and 12%) vs. 10 and 3 (77% and 23%) in Groups A and B, respectively (*p* = 0.41).

Group B was more commonly affected by stage ≥ 4 CKD, atrial fibrillation and reduced left ventricular ejection fraction, compared to patients of Group A. Pulmonary artery systolic pressure tended to be higher in Group B (*p* = 0.07). Panel reaction antibodies (PRA) > 20% were reported in 29% and 31% in patients of Groups A and B, respectively (*p* = 0.89).

The main cause of late re-HTx was grade ≥ 2 CAV, which developed after a median time of 12 years (IQR 7–14 years) in Group A and 13 years (IQR 10–19 years) in Group B patients (*p* = 0.13). The median time from grade ≥ 2 CAV diagnosis and re-HTx was 3 years (IQR 1.5–4 years) vs. 10 years (IQR 5–12 years) for Groups A and B (*p* < 0.01).

### 3.2. Donor Data

The median donor age tended to be higher in Group B compared to Group A patients (47 vs. 37 years, *p* = 0.07). The employment of extended-criteria donors was similar between the two groups (70% vs. 69%, *p* = 0.99) as well as the graft ischemic time and technique of graft preservation (Table 2).

### 3.3. Perioperative Outcome and Management

The perioperative results are reported in Table 3. Overall, 30-day mortality was 6.7% (n = 2), being 0% in Group A and 17% in Group B (*p* = 0.08). One patient, who required an arterio-venous extracorporeal life support (ECLS) implant, died of septic shock after one day of support, while another died of massive pulmonary hemorrhage; moderate to severe PGD developed in four Group A (23%) and six Group B (46%) (*p* = 0.19) patients. In Group B, another patient required ECLS and was successfully weaned after 5 days.

In seven patients, two of Group A (12%) and five of Group B (38%) (*p* = 0.08), re-HTx was combined with KTx. In four cases, KTx was performed during the same operation, after chest closure, and in three cases, it was performed within 24 h from re-HTx. In five patients with double transplant, temporary renal replacement treatment was required, and one died within 30 days because of sepsis. The incidence of renal replacement treatment was 23% and 54% in Group A and B recipients (*p* = 0.08).

At 1 year after re-HTx, the rates of grade ≥ 2 rejection episodes and infections requiring treatment were 29% and 41% vs. 31% and 38% in Groups A and B, respectively (*p* = 0.99 and *p* = 0.89).

### 3.4. Late Outcomes

The median follow-up was 74 (17–150) and 65 (3–111) months (*p* = 0.13) in Groups A and B, corresponding to 112 and 61 patient-years, respectively. There were 10 late deaths: 7 in Group A because of multiorgan failure (MOF, n = 3), severe acute rejection (n = 1), cerebrovascular accident (n = 1), sudden death (n = 1), and coronavirus infection (n = 1), and 3 in Group B because of MOF (n = 1), metastatic melanoma (n = 1), and severe acute rejection (n = 1). Grade ≥ 2 CAV occurred in two patients of Group A and one patient of Group B, 7, 8 and 12 years after re-HTx; all had focal coronary stenoses treated with percutaneous angioplasty.

Malignancies occurred in two patients of Group A (12%) and in three (27%) of Group B, corresponding to an incidence of 1.8 and 4.9%/patient-years (*p* < 0.01), respectively. In Group A, one case of lymphoproliferative disease after 2 years, which was treated with radiotherapy, and one case of renal cancer after 3 years requiring partial nephrectomy were observed; in Group B, three skin cancers (metastatic melanoma after 2 years in one patient and squamous carcinomas after 4 and 5 years in two) were treated with surgical excision. In all, Mycophenolate Mofetil was replaced with Everolimus, and the Cyclosporine or Tacrolimus dosage was lowered.

Out of the 11 patients who required renal replacement treatment immediately after re-HTx, 10 were discharged without need of hemodialysis during follow-up. One patient who previously had two KTx underwent re-HTx, followed after 20 months by a third KTx.

**Figure 1:**
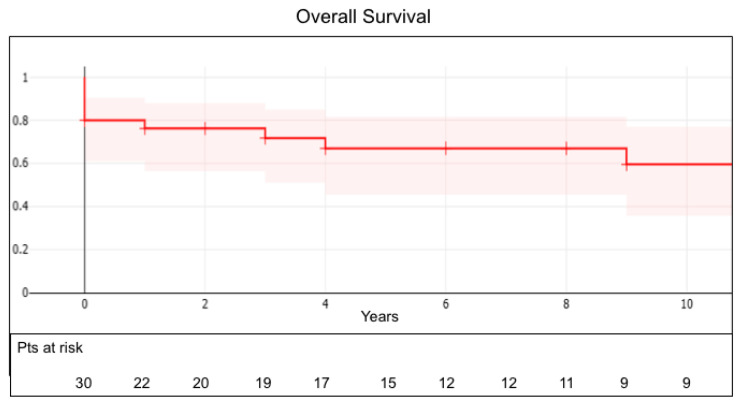
Actuarial survival in the entire population.

**Figure 2:**
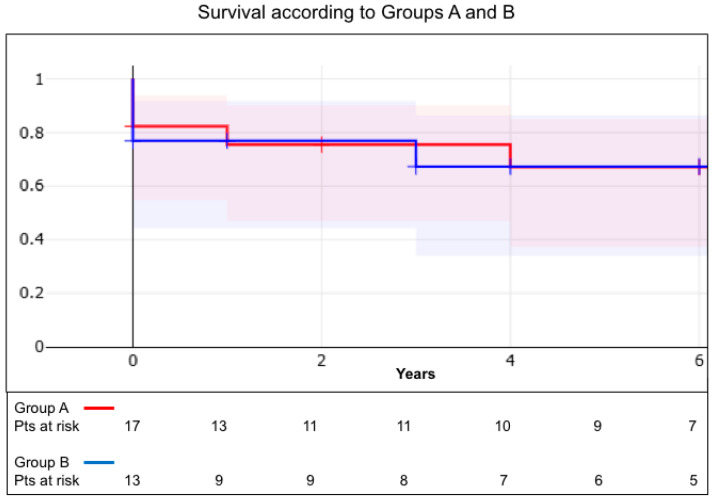
Actuarial survival in Group A and B patients.

Overall survival was 80 ± 7%, 68 ± 9% and 60 ± 11% at 1, 5 and 10 years (Figure 1); at 1 and 5 years, survival was 82 ± 9% and 68 ± 12% in Group A and 78 ± 11% and 67 ± 14% in Group B (*p* = 0.59), as reported in Figure 2.

According to multivariable analysis, only recipient age was independently associated with mortality (HR 1.07 [95% confidence interval between 1.001 and 1.14], *p* = 0.05), as shown in Supplementary Material S2.

### 3.5. Immunosuppressive Management

Maintenance immunosuppressive treatment before re-HTx consisted of Cyclosporine or Tacrolimus in 23 (77%) and 7 (23%) cases, combined with Everolimus (n = 25, 83%), Mycophenolate Mofetil (n = 3, 10%) or prednisone (n = 2, 7%) in Groups A and B.

Despite chronic immunosuppression, an induction treatment with Anti-Thymocyte Globulins for a maximum of 3 days was employed in 27 patients (90%), while in 3 patients, it was avoided due to lymphopenia (n = 1) and suspected infection.

While in 3 patients it was avoided due to lymphopenia (n = 1) and suspected infection during ECLS support (n = 1) in Group A and history of post-HTx lymphoproliferative disease (n = 1), in Group B. After re-HTx, Mycophenolate Mofetil was generally adopted (n = 30), while Cyclosporine was preferred over Tacrolimus in 28 patients because of the easier titration, especially in patients with severe renal impairment. The monitoring and treatment of rejection episodes followed our established protocol [19].

### 3.6. Incidence of Complications in Relation to Primary HTx Versus Re-HTx

The baseline clinical characteristics of the recipients at the time of primary HTx and of re-HTx are reported in Supplementary Material S1. In brief, at re-HTx, recipients were more commonly affected by CKD stage ≥4 and more commonly treated with renal re-placement therapy. At primary HTx, the rate of previous cardiac surgery was 40%.

Comparing primary HTx with re-HTx in the same population, the rate of grade ≥ 2 rejection episodes was 17% (n = 5) vs. 30% (n = 9) (*p* = 0.14) and the rate of infections 20% (n = 6) vs. 40% (n = 12) (*p* = 0.09).

Considering 516 patient-years for primary and 173 patient-years for re-HTx, the overall incidence of malignancies after primary vs. re-HTx was 0.58 vs. 2.89%/patient-years (*p* < 0.01).

Grade ≥ 2 CAV affected 25 patients (83%) after primary HTx, within a median time of 13 years (IQR of 9–15); only 3 of 28 discharged patients (11%) developed grade ≥ 2 CAV, 7, 8 and 12 years after re-HTx.

At 5 and 10 years, the survival free from grade ≥ 2 CAV was 93 ± 4% and 73 ± 8% vs. 93 ± 6% and 85 ± 10% for primary HTx and re-HTx, respectively (*p* = 0.13).

## 4. Comment

Re-HTx continues to be a controversial therapeutic option for patients affected by severe graft failure. The donor shortage, the increasing number of patients waiting for a primary HTx, and the initially reported poor outcomes have raised ethical concerns about its opportunity. As a result, re-HTx represents only 2–4% of all HTx performed annually in Western countries [2,3].

The timing and indications for re-HTx have been extensively related to outcomes, since primary graft failure and acute rejection have shown worse outcomes than CAV and chronic graft dysfunction [7,8,9,10,11,20].

Re-HTx as rescue therapy for early graft dysfunction was adopted in our early experience, but it is no longer considered as the treatment of choice for graft failure due to severe acute rejection because of poor outcomes. In such cases, we try to stabilize the hemodynamic status using short-term mechanical circulatory support, allowing the graft to recover. Furthermore, an indication for re-HTx should be reserved for patients with severe CAV and chronic graft dysfunction in the absence of active rejection [10,21,22].

Data reporting the results of re-HTx in patients after over a decade from primary HTx and, therefore, subjected to the effects of long-standing immunosuppression are quite limited, since most reports limit their data to the first or second post-HTx decades. In fact, studies derived from the United Network for Organ Sharing (UNOS) [10] and the Spanish Heart Transplant Registry [11] reported a median interval time from primary HTx to “late re-HTx” of 9.4 years (IQR 5.7–14.0) and 9.1 years (IQR 5.7–13.1), respectively. For such reasons, in reporting our experience with late re-HTx, we have further focused our attention on patients undergoing re-HTx within and beyond 20 years from the initial HTx. Our results confirm the satisfactory outcome of late re-HTx, which seems not to be impaired even if the post-HTx evaluation is extended ≥ 20 years.

Accordingly, this study provides interesting data on the clinical characteristics of patients requiring re-HTx after such a long period. In Group A, the time interval between the diagnosis of severe CAV and re-HTx was shorter than that in Group B, indicating a more rapid onset of symptomatic graft failure, which could be due to a more aggressive individual immune response, although this hypothesis needs to be supported by more consistent data. In these patients, CAV led more rapidly to a restrictive form of graft failure, due to stiffness of the ventricular walls, with maintenance of normal or close-to-normal LVEF, as also observed by others [23]. Instead, in Group B patients, who were more likely to have a reduced LVEF, atrial fibrillation and higher pulmonary artery pressure compared to Group A, graft failure due to CAV developed more progressively, leading to the onset of symptoms only when systolic graft function became severely compromised. This also seems to be confirmed by the higher rates of grade ≥4 CKD, infections and combined re-HTx and KTx in this group, possibly due to prolonged exposure to immunosuppression.

Early postoperative care is challenging in re-HTx patients, as demonstrated by the high rates of prolonged mechanical ventilation and renal replacement treatment observed in the current series. Moreover, due to the paucity of available data, there is no unanimous consensus on indications for and the management of post-re-HTx immunosuppressive therapy [22,23,24]. In our institution, early postoperative induction with anti-thymocyte antibodies represents a standard treatment aiming to provide effective immunosuppression, allowing at the same time slower titration to target levels of calcineurin inhibitors, which could be particularly attractive for patients with chronic renal impairment.

At 1 year, we reported 29% and 31% grade ≥ 2 acute rejections and 41% and 38% rates of infections in Groups A and B, data similar to those previously reported [2,22]. However, the higher rates of infections observed in re-HTx compared to primary HTx (40% vs. 20%, *p* = 0.09) could suggest a combined effect of overexposure to immunosuppression early after re-HTx in a more aging population.

Despite the International Society for Heart and Lung Transplantation reporting similar rates of neoplasms after HTx and re-HTx [2], others have considered re-HTx patients at higher risk of malignancies [5]. In this series, five patients experienced malignancies during follow-up (2.89 events % patient-years), an incidence significantly higher than that reported after primary HTx (0.58%patient-years, *p* < 0.01) in the same population. Interestingly, the incidence of malignancies was 1.8 and 4.9%patient-years (*p* < 0.01) for Groups A and B. Since neoplasms are considered one major cause for mortality at 5 years from HTx [25], it could be speculated that the incidence of this complication could be increased in re-HTx, secondarily to the repeated induction treatment and a higher immunosuppressant level in the early post-HTx period. Given the higher rates of malignancies in Group B, the immunosuppressive induction strategy and target levels of immunosuppression should probably be reconsidered and the need for a careful oncological follow-up particularly stressed, especially considering the older age of this population.

The presence of CKD in these patients represents a further important issue. In this series, 12 patients with stage ≥4 CKD underwent re-HTx: 7 received combined re-HTx and KTx, while 5 received re-HTx only. KTx combined with re-HTx was preferred in clinically stable patients with generally good results since only one such patient required dialysis during follow-up. Our findings further support concurrent KTx as an effective strategy for improving outcomes in re-HTx with severe renal failure, which must be taken into consideration after weighing the clinical conditions of each individual patient [26].

The 5-year freedom from severe CAV was similar after primary and re-HTx in the same patients even though the new grafts were exposed to a shorter period of immunosuppressive treatment. This apparent paradox might be explained only after a longer follow-up period, but most likely, other factors could be involved in CAV onset, besides the type and duration of therapy and time lapse, which should be more thoroughly investigated. One could argue that, after re-HTx, the probability of developing CAV could be lower than that of primary HTx, due to a prolonged immunosuppressive state. However, this hypothesis should be supported by a longer follow-up period.

The major limitation of this study is represented by its retrospective nature. However, this is a single-center study which is not biased by differences in patient selection or treatments which might be present when including patients from various institutions. Moreover, the number of patients is limited, but this reflects the experience of other single centers confirming that re-HTx has not yet received worldwide definitive acceptance. However, this issue limits the study’s power to draw firm conclusions, and, despite many comparisons approaching statistical significance, the findings obtained may serve to generate hypotheses for future research.

Also, data obtained from the comparison between primary and re-HTx were biased by the differences related to different stages of the patient’s clinical history and were not corrected for other variables. Thus, they should be interpreted with caution.

This study spans a long period of over 30 years, during which changes in surgical techniques and recipient management have occurred, which might have influenced patient outcomes. It must be noted, however, that, after 2000, for most re-HTx patients, management and protocols have remained the same.

The results of this study indicate that re-HTx could be considered an effective and feasible therapy for chronic graft dysfunction after HTx, also for patients more than 20 years after primary HTx. Exposure to immunosuppressive treatment for such a long time causes several degrees of end-organ injury, which leads to high rates of perioperative and long-term complications. In this setting, patient-tailored immunosuppressive management and close follow-up could represent the key to obtaining even more gratifying results. If confirmed by larger and prospective studies, listing for a re-HTx could be routinely considered in selected patients who develop late graft failure.

## Figures and Tables

**Table 1:** Recipients’ baseline characteristics.

Clinical Data	Group A (n = 17)	Group B (n = 13)	*p* Value
Years from first HTx, median (IQR)	15 (7–17)	23 (21–25)	<0.01
Age, years, median (IQR)	46 (42–58)	52 (46–58)	0.12
Male sex, n. (%)	12 (70)	8 (61)	0.60
Diabetes mellitus, n. (%)	4 (23)	1 (8)	0.37
Arterial hypertension, n. (%)	3 (18)	3 (23)	0.67
Dyslipidemia, n. (%)	6 (35)	3 (23)	0.41
Serum albumin (mg/dL), median (IQR)	39 (31–45)	40 (32–45)	0.88
BMI, median (IQR)	25 (21–29)	24 (20–28)	0.33
History of malignancy, n. (%)	1 (6)	2 (15)	0.45
CKD stages ≥ 4, n. (%)	4 (23)	8 (61)	0.04
RRT, n. (%)	3 (18)	6 (46)	0.19
Total bilirubin > 2 mg/dL, n. (%)	3 (18)	1 (8)	0.42
PRA > 20%, n. (%)	5 (29)	4 (31)	0.89
sPAP (mmHg), median (IQR)	31 (24–35)	38 (30–41)	0.07
Atrial fibrillation, n. (%)	2 (12)	6 (46)	0.03
Right ventricular dysfunction, n. (%)	4 (23)	5 (38)	0.44
TVR ≥ moderate, n. (%)	3 (18)	3 (23)	0.89
LVEF ≥ 50%, n. (%)	10 (59)	3 (23)	0.05
Inotropic support, n. (%)	6 (35)	2 (15)	0.22
IABP, n. (%)	1 (6)	0 (0)	0.99
ECLS, n. (%)	1 (6)	0 (0)	0.99
MV, n. (%)	1 (6)	0 (0)	0.99
Indication for re-HTx			0.24
CAV, n. (%)	13 (76)	12 (92)	
Chronic graft dysfunction, n. (%)	4 (24)	1 (8)	*p* value

HTx = Heart transplantation; IQR = Interquartile range; BMI = Body mass index; CKD = Chronic kidney disease; RRT = Renal replacement treatment; PRA = Panel reaction antibodies; sPAP = Systolic pulmonary artery pressure; TVR = Tricuspid valve regurgitation; LVEF = Left ventricular ejection fraction; IABP = Intra-aortic balloon pump; ECLS = Extracorporeal life support; MV = Mechanical ventilation; CAV = Cardiac allograft vasculopathy; re-HTx = Heart re-transplantation.

**Table 2:** Donor and procedural data.

	Group A (n = 17)	Group B (n = 13)	*p* Value
Age, years, median (IQR)	37 (23–50)	47 (34–54)	0.07
Age > 55 years, n. (%)	3 (18)	4 (31)	0.33
Male sex, n. (%)	10 (59)	7 (54)	0.78
Cause of death			
Cerebrovascular accident, n. (%)	7 (41)	7 (54)	0.49
Trauma, n. (%)	7 (41)	4 (31)	0.56
Other, n. (%)	2 (12)	2 (15)	0.88
Extended-criteria donor, n. (%)	12 (70)	9 (69)	0.93
Downtime ≥ 20 min, n. (%)	4 (23)	3 (23)	0.99
LVPWT > 12 mm, n. (%)	2 (12)	1 (8)	0.71
CAD, n. (%)	3 (18)	1 (8)	0.42
Diabetes mellitus, n. (%)	1 (6)	1 (8)	0.84
Drug abuse, n. (%)	1 (6)	0 (0)	0.21
Graft preservation			
Ice-cold storage, n. (%)	11 (65)	9 (69)	0.79
PSP preservation, n. (%)	2 (12)	2 (15)	0.77
OCS preservation, n. (%)	4 (23)	2 (15)	0.58
Graft ischemic time			
>4 h, n. (%)	4 (23)	4 (31)	0.65
minutes, median (IQR)	204 (185–241)	193 (146–248)	0.88
CPB time, minutes, mean (SD)	206 (47)	217 (70)	0.31

IQR = Interquartile range; LVPWT = Left ventricular posterior wall thickness; CAD = Coronary artery disease; PSP = Paragonix sherpapak; OCS = Organ Care System; CPB = Cardiopulmonary bypass.

**Table 3:** Post re-HTx outcomes.

	Group A (n = 17)	Group B (n = 13)	*p* Value
30-day mortality, n. (%)	0 (0)	2 (15)	0.08
Moderate PGD, n. (%)	4 (23)	5 (38)	0.37
Severe PGD, n. (%)	0 (0)	1 (8)	0.18
IABP, n. (%)	1 (6)	3 (23)	0.17
ECLS, n. (%)	0 (0)	2 (15)	0.08
MV > 72 h, n. (%)	7 (41)	3 (23)	0.29
RRT, n. (%)	4 (23)	7 (54)	0.08
Re-exploration for bleeding, n. (%)	4 (23)	3 (23)	0.99
ICU stay, days, median (IQR)	8 (5–20)	13 (8–30)	0.07
1-year ≥ 2 ACR or AMR episodes, n. (%)	5 (29)	4 (31)	0.99
*AMR episodes, n. (%)*	*1 (6)*	*1 (8)*	
*Mixed rejection, n. (%)*	*0 (0)*	*1 (8)*	
1-year treated infection, n. (%)	7 (41)	5 (38)	0.89
Combined kidney transplantation, n. (%)	2 (12)	5 (38)	0.08

PGD = Primary graft dysfunction; IABP = Intra-aortic balloon pump; ECLS = Extracorporeal life support; MV = Mechanical ventilation; RRT = Renal replacement treatment; ICU = Intensive care unit; IQR = Interquartile range; ACR = Acute cellular rejection; AMR = Antibody-mediated rejection.

## Data Availability

Data available on request due to privacy/ethical restrictions.

## Supplementary Materials

The following supporting information can be downloaded at: https://www.mdpi.com/article/10.3390/jcm15020564/s1, Supplementary Material S1: Baseline recipients’ characteristics at primary HTx and at re-HTx; Supplementary Material S2: Univariable and Multivariable analysis for survival.
