# Supplementary material for: Are the Results of Late Heart Re-Transplantation Influenced by the Time Interval from Primary Transplantation?
Source: J Clin Med. 2026 Jan 10;15(2):564. doi: 10.3390/jcm15020564 (PMC12842072; doi:10.3390/jcm15020564)
Supplement: Supplementary file 1 [file jcm-15-00564-s001.zip › jcm-4056577-supplementary.pdf]

MDPI

Journal of Clinical Medicine

Supplementary Material S1. Baseline recipients' characteristics at primary HTx and at re-HTx.

Clinical Data	HTx (n=30)	re-HTx (n=30)	p value
Age, years, median(IQR)	32 (25-42)	49 (44-58)	< 0.01
Diabetes mellitus, n.(%)	3 (10)	5 (17)	0.44
Redo cardiac surgery, n (%)	12 (40)	30 (100)	< 0.01
Arterial hypertension, n.(%)	4 (13)	6 (20)	0.49
Dyslipidemia, n.(%)	7 (23)	9 (30)	0.56
BMI, median (IQR)	24 (21-26)	25 (20-28)	0.68
CKD stages ≥4, n.(%)	5 (17)	12 (40)	0.04
RRT, n.(%)	2 (7)	9 (30)	0.02
Total bilirubin >2 mg/dl, n.(%)	2 (7)	4 (13)	0.39
sPAP (mmHg), median(IQR)	31 (24-35)	34 (26-38)	0.12
Inotropic support, n.(%)	6 (20)	8 (27)	0.54
IABP, n.(%)	1 (3)	1 (3)	0.99
ECLS, n.(%)	1 (3)	1 (3)	0.99
MV, n.(%)	1 (3)	1 (3)	0.99

HTx=Heart transplantation; re-HTx=heart re-transplantation; IQR=Interquartile range; BMI=Body mass index; CKD=Chronic kidney disease; RRT=Renal replacement treatment; sPAP=Systolic pulmonary artery pressure; IABP=Intra-aortic balloon pump; ECLS=Extracorporeal life support; MV=Mechanical ventilation.

 https://doi.org/10.3390/jcm15020564

Supplementary Material S2. Univariable and Multivariable analysis for survival

Variable	HR	95%CI	p value
Univariable analysis			
Group B	1.33	0.4-4.4	0.64
Recipient age	1.07	1.001-1.48	0.03
sPAP	1.61	0.44-7.38	0.41
CKD stages ≥4	1.78	0.46-6.9	0.4
Multivariable analysis			
Group B	0.89	0.16-4.77	0.89
Recipient age	1.07	1.001-1.14	0.05
sPAP	1.34	0.34-5.62	0.67
CKD stages ≥4	1.46	0.36-4.97	0.59

HR= Hazard ratio; CI= Confidence interval; sPAP=Systolic pulmonary artery pressure; CKD=Chronic kidney disease.

Disclaimer/Publisher's Note: The statements, opinions and data contained in all publications are solely those of the individual author(s) and contributor(s) and not of MDPI and/or the editor(s). MDPI and/or the editor(s) disclaim responsibility for any injury to people or property resulting from any ideas, methods, instructions or products referred to in the content.

https://doi.org/10.3390/jcm15020564
